# Apatinib for advanced sarcoma: results from multiple institutions’ off-label use in China

**DOI:** 10.1186/s12885-018-4303-z

**Published:** 2018-04-06

**Authors:** Lu Xie, Wei Guo, Ye Wang, Taiqiang Yan, Tao Ji, Jie Xu

**Affiliations:** 1 Peking University People’s Hospital, Peking, China; 2 Peking University Shougang Hospital, Peking, China; 3 Peking University International Hospital, Peking, China

**Keywords:** Apatinib, Tyrosine-kinase inhibitor, Osteosarcoma, Chondrosarcoma, Soft-tissue sarcoma, Ewing sarcoma

## Abstract

**Background:**

Anti-angiogenesis Tyrosine kinase inhibitors (TKIs) have been proved to show promising effects on prolonging progression-free survival (PFS) for advanced sarcoma after failure of standard multimodal Therapy. Methylsulfonic apatinib is one of those TKIs which specifically inhibits VEGFR-2. This paper summarizes the experience of three Peking University affiliated hospitals in off-label use of apatinib in the treatment of extensively pre-treated sarcoma.

**Methods:**

We retrospectively analysed files of patients with advanced sarcoma not amenable to curative treatment, who were receiving an apatinib-containing regimen between June 1, 2015 and December 1, 2016. Fifty-six patients were included: 22 osteosarcoma, 10 Ewing’s sarcoma, 3 chondrosarcoma and 21 soft tissue sarcoma.

**Results:**

With median follow-up time of 6 months (range, 0.7–18.0 m), thirty-five (62.5%) patients had partial response, and disease was stable in 11 (19.6%). The 4-month and 6-month progression-free survival rates were 46.3 and 36.5%, respectively. The median duration of response was 3.8 months (95% CI 1.9–5.6 m), with much variability among disease subtypes. The median overall survival was 9.9 months (95% CI 7.6–12.2 m). Grade 3 and 4 toxicities were observed in 8 (14.3%) patients, the most common being hypertension, pneumothorax, wound-healing problems, anorexia, and rash or desquamation.

**Conclusions:**

Apatinib might be effective, with a high objective response rate, in an off-label study of sarcoma patients with advanced, previously treated disease. The duration of response was consistent with reports in different subtypes of sarcomas. Prospective trials of apatinib in the treatment of selected subtypes of sarcomas are needed.

**Trial registration:**

Retrospectively registered in the Medical Ethics Committee of Peking University People’s Hospital, Peking University Shougang Hospital and Peking University International Hospital. The trial registration number is 2017PHB176–03 and the date of registration is January 20th 2017.

## Background

Sarcomas are a rare, heterogeneous family of mesenchymal tumors, consisting mostly of bone tumors and soft tissue sarcoma (STS) [1, 2]. Use of traditional chemotherapeutic treatments has been limited by poor response rates in patients with relapsed or advanced disease. Nowadays more and more attention are paid to anti-angiogenesis tyrosine kinase inhibitors (TKIs), especially in the field of advanced osteosarcoma and soft tissue sarcoma. However, no or little progress has been made in treatment of these tumors since Grignani et al. [3] reported landmark phase II cohort trials of sorafenib or sorafenib combined with everolimus [4] in advanced refractory osteosarcoma.. PALETTE study proved that pazopanib could obviously prolong the progression-free survival (PFS) by 3 months but the partial response rate was only 6%. [5]

China has many patients with advanced sarcoma who need to be treated and managed properly. However, the country lacks resources necessary for participation in large multi-center trials. Thus, based on the results of prospective trials abroad, patients with advanced and refractory metastatic disease here are often treated with apatinib off-label, which is also an anti-angiogenesis TKIs domestically made and highly selectively inhibitor on VEGFR-2.. The IC_50_ of apatinib is 2 nM for VEGFR-2, 70 nM for VEGFR-1, 420 nM for c-kit and 537 nM for PDGFR-β [6, 7].

This report aims to describe objectively the use, efficacy, and safety of apatinib in advanced sarcoma patients who have been previously treated in the orthopedic oncology departments of three affiliated hospitals of Peking University,in China: Peking University People’s Hospital, Peking University Shougang Hospital, and Peking University International Hospital. A determination will be made whether apatinib warrants further investigations for sarcoma patients.

## Methods

From June 1st 2015 to December 1st 2016, patients who met the following criteria were included: 1) histologically confirmed high-grade sarcoma; 2) initial treatment in the orthopedic oncology departments of the three affiliated hospitals of Peking University; 3) tumors not amenable to curative treatment or inclusion in clinical trials; 4) unresectable local advanced lesions or multiple metastatic lesions that could not be cured by local therapy; 5) measurable lesions according to Response Evaluation Criteria for Solid Tumors (RECIST1.1) [8]; 6) Eastern Cooperative Oncology Group performance status 0 or 1 [9]; and 7) acceptable hematologic, hepatic, and renal function.

All patients or children’s legal parent had ever signed informed consent for data collection and use for research purpose. The study was approved by the Institutional Review Board of Peking University People’s Hospital, Peking University Shougang Hospital, and Peking University International Hospital Ethics Committee for Clinical Investigation.

Because of various characteristics of diseases, we usually gave patients the following treatment before apatinib. Osteosarcoma patients usually progressed through four drugs, including doxorubicin, cisplatin, high-dose methotrexate, ifosfamide Ewing’s sarcoma patients usually progressed through at least two lines of chemotherapy, including VDC-IE (vincristine, doxorubicin, cyclophosphamide sequenced with ifosfamide and etoposide) and VTI (vincristine, temozolomide, irinotecan). For soft tissue sarcoma, patients usually progressed through at least doxorubicin and ifosfamide. But sometimes apatinib together with GT, which was gemcitabine 1000 mg/m^2^ d_1,8_ and docetaxel 75 mg/m^2^ d_8_ once every 21 day, were given to some initial ASPS and epithelioid sarcoma patients because of their poor response to conventional chemotherapy (Tables 1 and 3).

**Table 1:** population characteristics

Characteristics	Number of patients	Percentage & range	*P* (Cox analysis for PFS)
Gender	56	100%	*0.050*
Male	33	58.9%	
Female	23	41.1%	
Age at diagnosis			*0.982*
Median (min–max) (year)	24.5	9–63	
Pathological diagnosis	56	100%	*0.087*
Osteosarcoma	22	39.3%	
Ewing sarcoma	10	17.9%	
Synovial sarcoma	6	10.7%	
MPNST^a^	3	5.4%	
Epithelioid sarcoma	2	3.6%	
UPS^b^	4	7.1%	
Fibrosarcoma	1	1.8%	
Chondrosarcoma	3	5.4%	
ASPS^c^	3	5.4%	
Others^d^	2	3.6%	
Tumor grade			
Grade III	56	100%	
Location of primary disease	56	100%	*0.374*
Axial skeleton	17	30.3%	
Extremities	37	66.1%	
Others^e^	2	3.6%	
Localization of relapse	56	100%	*0.541*
Localized	3	5.6%	
Metastatic	41	73.2%	
Both	12	21.4%	
Type of metastasis	53	94.6%	*0.197*
Lung only	40	71.4%	
Bone only	3	5.4%	
Both	5	8.9%	
Other^e^	5	8.9%	
Time interval from initial chemotherapy to using apatinib			*0.584*
Median (min–max) (month)	15.6	0.9–373.9	
Number of previous treatment lines	56	100%	*0.231*
0	5	8.9%	
1	37	66.1%	
2	12	21.4%	
> 2	2	3.6%	
Follow-up time			
Median (min–max) (month)	6.0	0.7–18.0	

^a^MPNST: malignant peripheral nerve sheath tumor

^b^UPS: undifferentiated pleomorphic sarcoma

^c^ASPS: alveolar soft part sarcoma

^d^others including extraskeletal osteosarcoma one case and mucinous type liposarcoma one case

^e^others including mediastinum and soft tissue of the backside

^f^others including lymph nodes metastasis or intravenous tumor emboli as well as liver, brain metastasis

In the phase I trial, apatinib (Jiangsu Hengrui Medicine, Lianyungang, China) had good oral bioavailability at a dose of 850 mg a day, the maximum-tolerated dose [10]. Our patients were mostly given 750 mg apatinib orally once daily for body surface area (BSA) > 1.5, and 500 mg daily for BSA < 1.5. If the patient was less than 10 years of age, we usually used 250 mg directly. If treatment interruptions occurred because of grade 3 hematologic or grade 2 non-hematologic toxicities, doses were reduced, and supportive care was given for the management of adverse events (AEs).

The primary objective of this study was to summarize our experience on the efficacy of off-label use of apatinib in sarcoma patients. Our main concern was the objective response rate (CR + PR) and progression-free-survival (PFS) for each protocol as described containing apatinib according to RECIST 1.1. Together with that, overall survival (OS), duration of response (DR) and the characterization of toxicities were also described. In our retrospective study, PFS was defined as time from the start of using apatinib until disease progression or death, whichever occurred first. The time from appearance of response or stable disease to progression or death was thus considered the DR.

PFS and OS were estimated by use of the Kaplan Meier method, with 95% confidence interval (CI), and comparisons were made with a log-rank test in the IBM SPSS 22.0 software. Safety evaluation was based on the frequency and severity of toxicities, graded according to the Common Terminology Criteria for Adverse Events [11]. Quantitative variables and categorical variables were analyzed with Cox univariate analysis. All statistical analyses were two-sided, and significance was set at *P* < 0.05 or at the 95% CI for the results of statistical tests. The database was locked for statistical analysis in January 2017, and this is a descriptive analysis.

## Results

### Patients’ characteristics

From June 1st 2015 to December 1st 2016, 63 consecutive advanced sarcoma patients were registered. Median follow-up time was 6.0 months (range, 0.7–18.0 m). Five patients were lost to follow up; 1 patient stopped using apatinib because of toxicity and another dropped out for another reason. Finally, 56 patients were enrolled: 22 osteosarcoma, 10 Ewing’s sarcoma, 6 synovial sarcoma, 3 malignant peripheral nerve sheath tumors (MPNST), 2 epithelioid sarcoma, 4 undifferentiated pleomorphic sarcoma (UPS), 1 fibrosarcoma, 3 chondrosarcoma, 3 alveolar soft part sarcoma (ASPS), 1 extraskeletal osteosarcoma, and 1 mucinous-type liposarcoma (Table 1). Seventeen (30.3%) of the sarcomas originated primarily from the axial skeleton; 37 (66.1%) from extremities; 2 (3.5%) were soft tissue sarcomas originated from the mediastinum or back side. Forty (71.4%) patients had only multiple pulmonary metastasis; 3 (5.4%) had only multiple bone lesions; 5 (8.9%) had metastasis of both lung and bone; and 5 (8.9%) had metastases to other sites. Table 1 illustrates that none of the clinicopathological factors examined (gender, age, pathological subtypes, location of primary disease, localization of relapse, type of metastasis, time interval from initial chemotherapy to starting using apatinib, number of previous treatment lines) had an evident influence on progression-free survival (PFS) (*P* ≥ .05).

Before treatment with apatinib, a median of 1.5 lines of chemotherapy (range 1–4) was administrated. Five (8.9%) patients received no chemotherapy before using target therapy, 3 of whom had ASPS and 2 had epithelioid sarcoma. Thirty-seven (66.1%) patients (mostly osteosarcoma) had progressed through 1 line of chemotherapy before using apatinib, while 14 (25%) had been through 2 or more than 2 lines of chemotherapy (Table 1).

### Treatment protocols

Forty-four of the 56 (78.6%) patients received only apatinib (oral administration); 7 (12.5%) received apatinib and everolimus in combination; and 5 (8.9%) received apatinib with gemcitabine and docetaxel (Table 2).

**Table 2:** Different treatment combination and median duration of response

Target therapy	Patient number (N)	Best response^a^	Median (average) DR (months)
Apatinib alone	44 (78.6%)	PR	3.8 (5.4)
Apatinib+everolimus	7 (12.5%)	PR	8.5 (7.3)
Apatinib+GT^b^	5 (8.9%)	PR	8.5 (7.3)

^a^*PR* partial response, *SD* stable disease according to RECIST 1.1

^b^*GT* chemo-protocol combined with gemcitabine 1000 mg/m^2^ d_1,8_ and docetaxel 75 mg/m^2^ d_8_ once every 21 days

Most of our patients were conventionally evaluated by their doctors at clinic every 2 months with at least chest CT and imaging of tumor lesions at other sites. If some of them could not go to clinic because of poor health status, our medical secretaries would call the patients for updates. However at last information collection, 5 patients were lost to follow-up (we usually defined as no information update for at least three months). Eventually we reviewed all their radiographs and pathological materials for this study.

### Efficacy of apatinib-included therapies

**Fig. 1:**
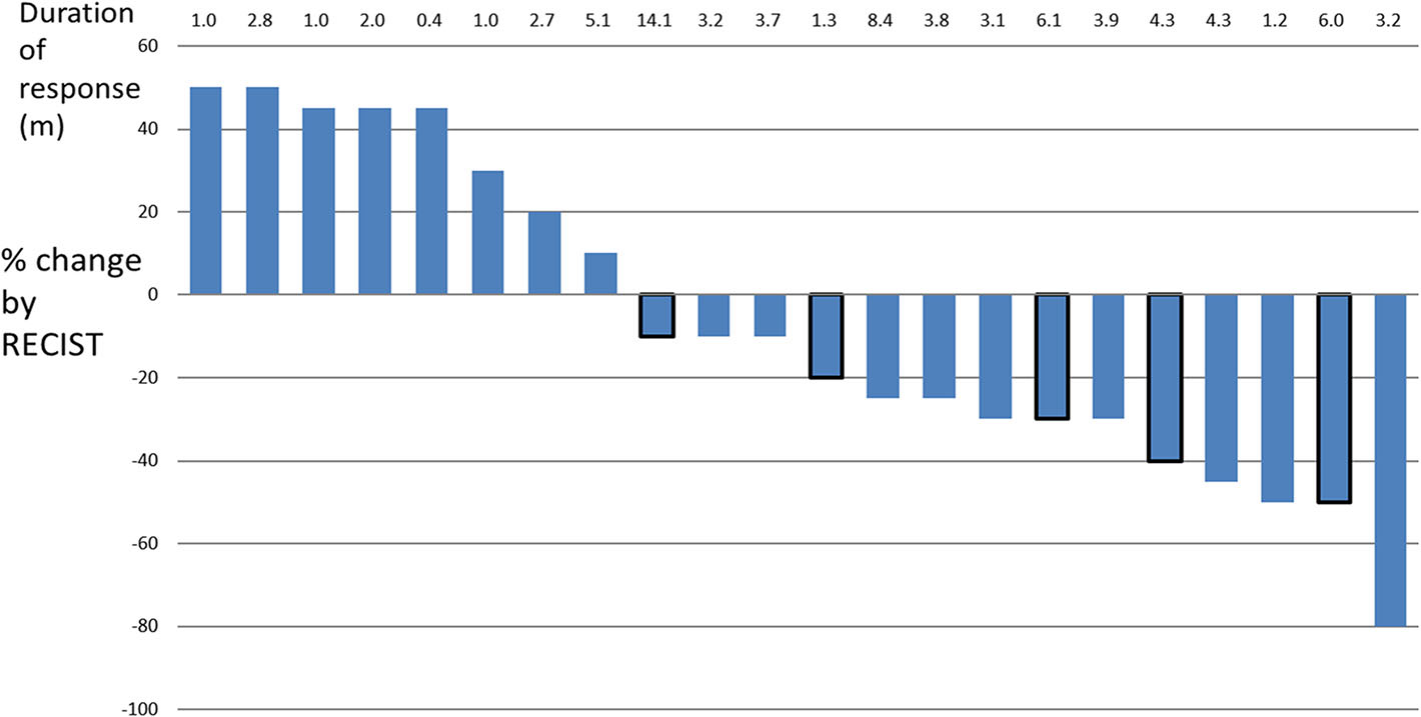
Waterfall plot of best change from baseline for 22 osteosarcoma patients. Patients’ clinical evaluations are indicated on the vertical graph as total volume increase or decrease. The numbers on the horizontal graph indicate the number of months of duration response. Strips with black frame indicate follow-up not yet at end point, and the patients’ status might continue unchanged for some while

As of the most recent follow up, 35 (62.5%) patients had partial responses and 11 (19.6%) had stable disease (Fig. 1). The 4-month and 6-month PFS rates were 46.3 and 36.5%, respectively. The median duration of response (DR) was 3.8 months (95% CI,; 1.9–5.6 m; which varied among pathological subtypes: 3.1 m (95% CI; 2.7–4.1 m) for osteosarcoma, 2.0 m (95% CI; 1.3–2.7 m) for Ewing’s sarcoma, 5.2 m (95% CI; 0.9–9.5 m) for synovial sarcoma, 8.8 m (95% CI; 4.3–11.5 m) for MPNST, and 5.6 m (95% CI, 1.3–9.8 m) for UPS (Table 3).

**Table 3:** Different disease and duration of response

Pathological diagnosis	Target therapy protocol	Patients number (N)	Best response^a^	Median (average) DR (months)
Osteosarcoma	Apatinib alone	22	PR	3.1 (3.7)
Ewing sarcoma	Apatinib + everolimus & apatinib alone	10	PR	1.5 (3.3)
Synovial sarcoma	Apatinib alone	6	PR	5.2 (5.8)
MPNST^c^	Apatinib alone	3	PR	8.8 (10.1)
Epithelioid sarcoma	Apatinib + GT^b^	2	PR	(4.7)
UPS^d^	Apatinib alone	4	PR	5.6 (5.0)
Fibrosarcoma	Apatinib alone	1	PR	2.7
Chondrosarcoma	Apatinib alone	3	PR	(7.4)
ASPS^e^	Apatinib + GT^b^	3	PR	(7.4)
Extraskeletal osteosarcoma	Apatinib alone	1	SD	6.6
Mucinous type liposarcoma	Apatinib alone	1	PD	1.0

^a^*PR* partial response, *SD* stable disease according to RECIST 1.1

^b^*GT* chemo-protocol combined with gemcitabine 1000 mg/m^2^ d_1,8_ and docetaxel 75 mg/m^2^ d_8_

^c^*MPNST* malignant peripheral nerve sheath tumor

^d^*UPS* undifferentiated pleomorphic sarcoma

^e^*ASPS* alveolar soft part sarcoma

**Fig. 2:**
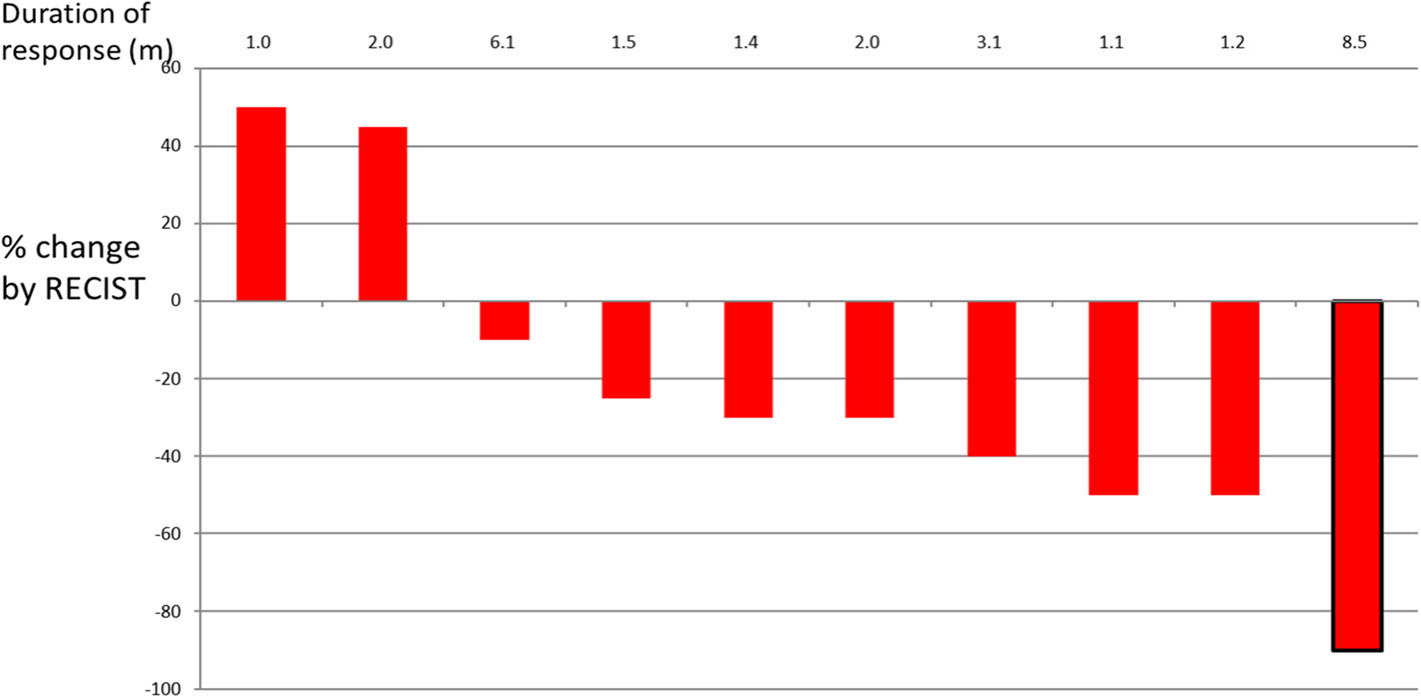
Waterfall plot of best change from baseline for 10 Ewing sarcoma patients

**Fig. 3:**
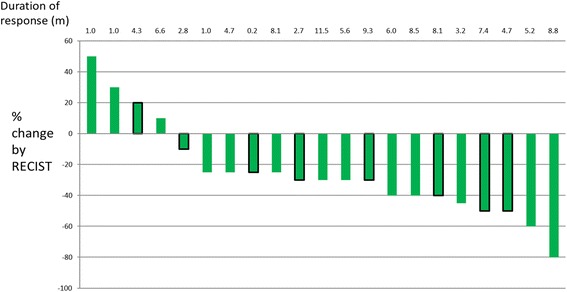
Waterfall plot of best change from baseline for 21 soft tissue sarcoma patients

The response conditions are illustrated in in Figs. 1, 2 and 3. The objective response rate (CR + PR according to RECIST 1.1) was 40.9% (9/22) for osteosarcoma, 70% (7/10) for Ewing’s sarcoma, 100% (3/3 cases) for chondrosarcoma, and 71.4% (15/21) for soft tissue sarcoma.

### Toxicity and safety

Treatment was interrupted in 10/56 (18.0%) cases because of disease progression. A 16-year-old female osteosarcoma patient died of cancer because of embolism of pulmonary venous tumor into the middle cerebral artery, and a 21-year-old male osteosarcoma patient had a seizure-like attack after taking apatinib 750 mg once daily for 3 days; the patient recovered gradually after stopping the drug. We had no explanation for this event except for a 3-week interval between stopping ifosfamide chemotherapy and starting apatinib; this rare scenario did not occur again in our series.

The adverse events are summarized in Table 4. Twenty-six Grade 3 or 4 events were recorded. Although the daily dose of apatinib we used was lower than that used in the phase II of apatinib treatment of metastatic gastric cancer [12], adverse events were not fewer, although the main kinds of adverse events were slightly different: most Grade 3 and 4 toxicities were hypertension, pneumothorax, wound-healing problems, anorexia, and rash or desquamation.

**Table 4:** Adverse Events

	Total N(%)	Grade		
		1	2	3–4
Apatinib alone^a^	45 (100%)			
Fatigue	8(17.8%)	5	2	1
Hypertension	35(77.8%)	27	3	5
Proteinuria	4(8.9%)	3	1	
Hand-foot syndrome	12(26.7%)	10	2	
Diarrhea	9(20%)	5	3	1
Weight loss	19(42.2%)	17	2	
Hair hypopigmentation	25(55.6%)	20	5	
Anorexia	17(37.8%)	10	4	3
Rash or desquamation	26(57.8%)	15	9	2
Mucositis	2(4.4%)	2		
Pneumothorax	9(20%)		3	6
Wound-healing problems	6(13.3%)		1	5
Elevated Aminotransferase or bilirubin	3(6.7%)	2	1	
Thrombocytopenia	7(15.6%)	5	1	1
Seizure-like attack	1(2.3%)			1
Pancreatitis	1(2.2%)	1		
Anemia	2(4.4%)	2		
Cranial neuritis	1(2.3%)	1		
Apatinib + everolimus^b^	7 (100%)			
Mucositis	7(100%)	2	4	1
Hypertension	4(57.1%)	2	2	
Rash or desquamation	5(71.4%)	2	3	
Gastrointestinal uncomfort	1(14.3%)		1	
Apatinib + GT^c^	5 (100%)			
Hypertension	1(20%)		1	
Rash or desquamation	2(40%)	2		
Wound-healing problems	1(20%)	1		
Thrombocytopenia	2(40%)	1	1	

^a^Apatinib alone: apatinib 500-750 mg/d according to the patient’s weight

^b^Apatinib + everolimus: apatinib 250–500 mg/d + everolimus 5 mg/d according to the patient’s weight

^c^*GT* chemo-protocol combined with gemcitabine 1000 mg/m^2^ d_1,8_ and docetaxel 75 mg/m^2^ d_8_ once every 21 days

## Discussion

In this study, we found an objective response rate with apatinib used off-label in refractory relapsed sarcoma (40.9%(9/22) for osteosarcoma, 70%(7/10) for Ewing’s sarcoma, and 71.4%(15/21) for soft tissue sarcoma). Also, except for osteosarcoma, the DR of other sarcomas was not inferior to that reported with other TKIs therapy, as shown in Table 5 [3, 4, 13–15]. In comparison with different combination of therapies, PFS seemed superior for apatinib together with sirolimus, but there was no statistically significant difference (*P = 0.12*). To determine apatinib’s effectiveness, the drug should be evaluated separately in treatment of various types of tumors.

**Table 5:** Previous studies about target therapy on sarcoma

Drug	Combined with chemotherapy	The first author’s last name	Year of publication	Trial Sponsor	Number of patients (N)	Clinical outcome
osteosarcoma						
	GT	Elizabeth Fox	2012	SARTCS^f^	14	ORR 7.1%;
Sorafenib	no	Grignani	2011	Italian Sarcoma Group	35	4 m-PFS^a^ 46%; DR^d^ 4 m; ORR^c^14%;
Trastuzumab	Cytotoxic Chemotherapy	Ebb	2012	COG^e^	41	30 m-EFS 32%; 30 m-OS^b^ 50%; without significant difference comparing with control group
Sirolimus	Cyclophosphamide	Schuetze	2012	Michigan University	5	ORR 0%; 4 m-PFS 30% (combined with other sarcoma)
Cixutumumab and Temsirolimus	no	Schwartz	2013	MSKCC^g^ fund	24	ORR 13%; median PFS 6w
Cixutumumab	no	Weigel	2014	COG	11	ORR 0%; 1/11 SD for 140 d
R1507	no	Pappo	2014	SARTCS^f^	38	ORR 2.5%; DR: 12w; 12w-PFS 17%
Sorafenib; Everolimus	no	Grignani	2015	Italian Sarcoma Group	38	6 m-PFS 45%; DR 5 m; ORR 10%
Cixutumumab; Temsirolimus	no	Wagner	2015	COG	11	ORR 0%;
Dasatinib	no	Schuetze	2016	SARTCS	46	ORR 6.5%; DR 5.7 m; 2y-OS 15%
Apatinib	no	Present study	2017		22	ORR 40.9%;median PFS 3.1 m; 4 m PFS 24.1%; 6 m PFS 18.1%
Ewing sarcoma						
	GT	Elizabeth Fox	2012	SARTCS	14	ORR 14.3%;
R1507	no	Pappo	2011	SARTCS	115	ORR 9.6%; median PFS 1.3 m; median OS 7.6 m
Figitumumab	no	Juergens	2011	European organization	106	ORR 14.2%; median PFS 1.9 m; median OS 8.9 m
Cixutumumab + temsirolimus	no	Schwartz	2013	MSKCC	27	ORR 14.8%; median PFS 7.5w; median OS 16.2 m
Olaparib	no	Choy E	2014	MGH^h^	12	ORR 0%; DR 5.7w;
Cixutumumab + temsirolimus	no	Wagner LM	2015	COG	43	ORR 0%; 12w-PFS 16%;
Apatinib+everolimus & apatinib alone	no	Present study	2017		10	ORR 70%; median PFS 2.0 m; 12w-PFS 22.5%
Soft tissue sarcoma						
	Topotecan +carboplatin	Bochennek K	2013	CSTSG^i^	34	ORR 38%;
Pazopanib	no	Winette T A	2012	EORTC^j^ and the PALETTE study group	246	ORR 6%; median PFS 4.6 m; median OS 12.5 m
Olaratumab	Doxorubicin	William D Tap	2016	MSKCC and 16 clinical sites in US	15 in IB trial and 67 in II trial	ORR 18.2%; median PFS 6.6 m; median OS 26.5 m
Regorafenib	no	Thomas Brodowicz	2015	International trial (France, Austria, Germany)	82	Median PFS 5.6 m for SS and 2.9 m for none specific;
Apatinib alone & apatinib+everolimus	Sometimes accompanied with GT^k^	Present study	2017		21	ORR 71.4%; median PFS 6.6 m; 4 m-PFS 71.4%; median OS 9.9 m
chondrosarcoma						
	GT	Elizabeth Fox	2012	SARTCS	25	ORR 8%;
GDC-0449	no	A. Italiano	2013	French Sarcoma Group/US; French National Cancer Institute	39	ORR 0%; median PFS 3.5 m; 6-m PFS 28.2%; 1-y PFS 19.2%
Imatinib	no	Grignani	2011	Italian Sarcoma Group	26	ORR 0%; 4 m-PFS 31%; median OS 11 m;
Sirolimus	cyclophosphamide	Bernstein-Molho R	2012	Israel	10	ORR 10%; 60% SD for more than 6 m; median PFS 13.4 m
IDH^l^ inhibitor	no	NCT02273739; NCT02481154; NCT02073994; NCT02496741	2016–2017			Under investigations
Apatinib alone	no	Present study	2017		3	ORR 100%; median PFS 7.37

^a^PFS: progression-free survival

^b^OS: overall survival

^c^ORR: overall response rate, defined as complete responses (CRs) + partial responses (PRs) + MRs;

^d^DR: Duration of response

^e^COG: Children’s Oncology Group

^f^SARTCS: Sarcoma Alliance for Research Through Collaboration Study

^g^MSKCC: Memorial Sloan-Kettering Cancer Center

^h^MGH: Massachusetts General Hospital

^i^CSTSG: Cooperative Soft Tissue Sarcoma Group

^j^EORTC: European Organization for Research and Treatment of Cancer

^k^GT: chemo-protocol combined with gemcitabine 1000 mg/m^2^ d_1,8_ and docetaxel 75 mg/m^2^ d_8_

^l^IDH: isocitrate dehydrogenase

Osteosarcoma patients whose disease relapses after failing standard chemotherapy present a challenging treatment dilemma. Some patients, through aggressive surgical resection of all gross disease, may have long-term survival [16]. The choice of second-line chemotherapy and the use of investigational drugs are not standardized, and the outcomes are dismal [17]. Maldegem et al. [15] summarized phase I/II clinical trials conducted between 1990 and 2010 in osteosarcoma and Ewing’s sarcoma; results were disappointing: only 8% CR, 2.8% PR, and 4% SD. Many active agents have been tested also in small series for treatment of osteosarcoma. Most anti-angiogenesis TKIs can only keep the tumor stable but not make it shrink [18]. The greatest progress in phase II trials belongs to the Italian Sarcoma Group; they have held 2 cohort phase II trials with advanced osteosarcoma patients and found an objective response rate (ORR) of 14 and 10% [3, 4]. However, 45% 6-month PFS (combination therapy) was less than the pre-specified threshold of activity deemed worthy of a phase III trial (6-month PFS of 50% or greater). In Table 5, apatinib had a higher rate of response than did sorafenib, but the duration of response seemed to be shorter.

In this study, we did notice this phenomenon that sometimes most or some patients’ lesions shrunk or remained stable during observation, while one or two lesions progressed. And this is especially common phenomenon happened during the third month after using apatinib for osteosarcoma. Patients might still get benefit from this VEGFR-2 highly selective drug with help of local therapy for those advanced lesion because of tumor heterogeneity. However as we use the criteria of RECIST 1.1, the duration of response seemed to be short. From our 56 patients, 4 osteosarcoma patients and one synovial sarcoma patient were in these circumstances.

Ewing’s sarcoma is genetically characterized by chromosomal translocation involving the Ewing’s sarcoma breakpoint region 1 (EWSR1) gene.. In this study we have 10 advanced Ewing’s sarcoma cases. Table 5 illustrates that for refractory Ewing’s sarcoma, the objective response rate was only 0 to 14.8% [15, 19, 20]. Nevertheless, the DR for Ewing’s sarcoma in various reports has been short compared with that of other sarcomas, with a median time of 5.7 weeks to less than 2 months [21–23]. In our study, more than half the Ewing’s sarcoma patients took apatinib together with everolimus, whereas the remainder took apatinib alone. Seventy percent of all these patients, who had apatinib containing protocol, had partial response, which seemed to indicate that apatinib was the most effective in these trials, and that anti-VEGFR2 target therapy might be another promising approach for treating Ewing’s sarcoma although with limited duration of response.

Soft tissue sarcoma is another huge group of sarcomas with diverse biological behaviors. For advanced cases, the only truly new treatment approved for sarcoma failing standard therapy is trabectedin, which has been approved by the European Medicines Agency 2007 [24]. Gemcitabine with dacarbazine or docetaxel [13, 25] and paclitaxel for treatment of angiosarcoma [26] seemed to improve PFS and OS in non-randomized and adaptively randomized trials. Targeted therapies, such as imatinib and sunitinib, have activity against gastrointestinal stromal tumors [27, 28]. Generally, anti-angiogenesis TKIs therapy with pazopanib has been a hallmark for all non-adipocytic soft tissue sarcoma after phase II and III trial verification, with median PFS 4.6 months (3.7–4.8 m; 95% CI) and best overall objective response rate of 6% (14/246) [14, 29]. Thomas et al. [30] reported that regorafenib, which is an inhibitor of VEGFR-1, − 2 and − 3 and tumor cell signaling kinases (RET, KIT, PDGFR, and Raf), yielded median PFS of 4.6 months in advanced sarcoma patients, which was almost the same as with pazopanib. Recently, the US Food and Drug Administration approved olaratumab [31], a human antiplatelet-derived growth factor receptor α monoclonal antibody, together with doxorubicin as first-line therapy for unresectable or metastatic soft tissue sarcoma. The approval was based on the drug having significant improvement in median OS (11.8 months), however this is for initially treated soft tissue sarcoma not for refractory cases. We used various combinations of therapy, including apatinib, achieving ORR of 71.4%, which is an astonishing result compared with other therapies [14, 24, 31] for treatment of advanced sarcoma. Although there were only 21 cases, we compared the subtype constitution in Table 3 and believed that it did not have obvious selective bias. In comparison with those agents, apatinib seemed to be more effective. The drug needs to be tested against other types of soft tissue sarcoma, such as MPNST and ASPS. We had 3 MPNST patients treated with apatinib, two of whom attained PR, and the PFS was 18.0 and 10.2 months. The other MPNST patient manifested as SD, and until last follow-up, at 4.3 months, her disease was stable. For target therapy for ASPS [29], objective response has rarely been reported, perhaps because the disease is indolent, progressing over decades, and few drugs have caused shrinkage of the tumors. However, with apatinib, which is a highly selective inhibitor of VEGFR-2, 2 of our 3 ASPS patients had PR, which seems a notable response. However, these PR cases firstly manifested as SD for 2 or 3 months and then started to shrink. However, the median OS with apatinib-containing therapy is shorter than that with pazopanib (9.9 m vs and 12.5 m). We suppose that this difference may be because patients with secondary resistance to apatinib might quickly die of the disease without much more efficient treatment options.

Our experience with toxicity associated with apatinib (Table 4) seemed to be more severe from that described in clinical trials [7]. One patient had to stop using apatinib because of neuro-toxicity. Three patients had so serious anorexia and weight loss that they stopped using the agent. Nine of our patients had treatment-related pneumothorax and six patients had wound healing problems.

We acknowledge this study’s limitations. First, it is a retrospective study that some patients might have some combination therapy which made this study not so suitable for comparion with other drugs. Second, because of the rarity of some types of sarcoma, we had insufficient numbers to permit subset analyses, which could have reduced the statistical power. Third, the study is off-label; hence, it may not have been as rigorously controlled as are prospective trials. Finally, 5 patients were lost to follow-up, which may have affected data accuracy.

## Conclusions

Apatinib might be with a high objective response rate, in an off-label study of sarcoma patients who had tumors not amenable to curative treatment or inclusion in clinical trials. The duration of response were consistent with responses reported in clinical trials with other anti-angiogenesis TKIs. Investigation of apatinib in the treatment of some special subtypes of sarcoma, for example metastatic MPNST and ASPS, in prospective trials is needed.

## Abbreviations

95% CI: 95% confidence interval
AEs: Adverse events
ASPS: Alveolar soft part sarcoma
BSA: Body surface area
CS: Chondrosarcomas
DR: Duration of response
EFS: Event-free survival
ES: Ewing’s sarcoma
ESFT: Ewing’s sarcoma family tumors
GT: Gemcitabine and docetaxel
IDH: Isocitrate dehydrogenase
IGF-1R: Insulin-like growth factor 1 receptor
MPNST: Malignant peripheral nerve sheath tumors
mTOR: Mammalian target of rapamycin
ORR: Object response rate
OS: Overall survival
PDGFR: Platelet-derived growth factor receptor
PFS: Progression-free survival
PR: Partial response
RECIST: Response Evaluation Criteria for Solid Tumors
SD: Stable disease
STS: Soft tissue sarcomas
TKIs: Tyrosine Kinase Inhibitors
UPS: Undifferentiated pleomorphic sarcoma
VDC-IE: Vincristine, doxorubicin, cyclophosphamide sequenced with ifosfamide, etoposide
VEGFR: Vascular endothelial growth factor receptor
VTI: Vincristine, temozolomide, irinotecan
